# The Post-Activation Potentiation Effects on Sprinting Abilities in Junior Tennis Players

**DOI:** 10.3390/ijerph19042080

**Published:** 2022-02-13

**Authors:** Luis Miguel Fernández-Galván, Pablo Prieto-González, Jorge Sánchez-Infante, Pedro Jiménez-Reyes, Arturo Casado

**Affiliations:** 1 Department of Physical Education, Sport, and Human Movement, Autonomous University of Madrid, 28049 Madrid, Spain; luisdepucela@gmail.com; 2 Health and Physical Education Department, Prince Sultan University, Riyadh 11586, Saudi Arabia; 3 Performance and Sport Rehabilitation Laboratory, Faculty of Sport Sciences, University of Castilla-La Mancha, 45071 Toledo, Spain; jorge.fisio.uclm@gmail.com; 4 Center for Sport Studies, Rey Juan Carlos University, 28032 Madrid, Spain; peterjr49@hotmail.com (P.J.-R.); arturo.casado@urjc.es (A.C.)

**Keywords:** post-activation potentiation, tennis, sprinting, acute performance, force-velocity profile

## Abstract

Objective: This study aimed to compare the acute effects of a full squat (SQ) or hip thrust (HT) with two different loading intensities (60% and 85% 1 RM) on sprint ability in junior male tennis players. Methods: Nineteen tennis players were included in this research. They underwent four different experimental conditions: HT at 60% 1 RM, HT at 85% 1 RM, SQ at 60% 1 RM, or SQ at 85%. The force–velocity (F–V) profile was used to assess tennis players’ sprint acceleration ability before and after applying the conditioning stimulus. The variables registered were as follows: 5 m test (5 m), 10 m test (10 m), maximum theoretical force (F_0_), maximum power (P_max_), and the maximal ratio of horizontal-to-resultant force (RF_peak_). Results: Significant improvements in 5 m, P_max_, and RF_peak_ were observed when the conditioning stimulus was performing one set of seven reps of HT at 60% 1 RM. When the activation protocol was one set of seven reps of SQ at 60% 1 RM, significant improvements in 5 m, 10 m, F_0_, P_max_ (N), and RF_peak_ were detected. Additionally, performing one set of three reps of SQ at 85% 1 RM as an activation protocol provided significant improvements in F_0_. *Conclusion*: The use of HT and SQ with a load of 60% 1 RM improved the sprint F–V profile components related to the acceleration phase of the sprint in junior tennis players. Using intensity loads of 85% 1 RM is not adequate to increase acute sprint performance in this population. HT presents a higher transferability to sprinting in the first 5 m of sprinting, whereas SQ provides acute improvements in different sprinting phases.

## 1. Introduction

Tennis is a complex racquet sport played by two opposing players or pairs that perform intermittent efforts. In tennis, players compete against one opponent in singles or two opponents in doubles who condition the motor actions of each player [1]. Tennis has significantly evolved during the last decades [2,3]. In addition to the well-known technical and tactical requirements, physical fitness is now also a relevant performance factor [3]. During the effective playing time, among all the technical skills and movements performed by the tennis players, serving, accelerations, and changes of directions are the key performance actions. Therefore, performance in this sport is largely conditioned by power, agility, and speed abilities [2,3,4,5].

In youth tennis, the internal and external loads slightly differ with respect to elite tennis [6]. Even so, it has been determined that explosive and ballistic actions are also key performance aspects in youth tennis [7]. Thus, junior tennis players, coaches, and physical trainers should aim to improve strength, power, and sprinting abilities. Additionally, as young athletes acquire a higher level of sports maturity, physical fitness training becomes increasingly relevant [8]. 

In this regard, one of the strategies currently implemented to attain acute increases in athletes’ functional performance in explosive and ballistic exercise is post-activation potentiation (PAP). PAP has been defined by Tillin and Bishop as the situation in which muscle performance is acutely improved as a result of a prior voluntary contraction [9]. Similarly, Gołaś et al. define PAP as an acute enhancement of performance or an enhancement of factors determining an explosive sports activity following a preload stimulus [10]. PAP enhances sports performance through the following mechanisms: (a) increased number of active motor units (motor unit recruitment), enhanced motor unit synchronization, decreased presynaptic inhibition, and increased number of nerve impulses transmitted [9,11]; (b) modifications in pennation angle; and (c) phosphorylation of myosin regulatory light chains [9,11]. After receiving the appropriate conditioning stimulus, performance optimization depends on the balance between fatigue and potentiation [12]. PAP depends on athletes’ training level, muscle fiber type, muscle contraction type and duration, and stimulus volume and intensity [13]. Thus, it has been verified that well-trained athletes respond better to PAP than recreational athletes. Subjects with a higher percentage of type II muscle fibers also obtain better PAP responses. Likewise, voluntary contractions provide better responses than electrostimulation. Additionally, it must be taken into account that there is a consistent and significant inter-individual variability in the responses obtained by the athletes after the implementation of PAP strategies. For this reason, it is not possible to establish the optimal PAP conditioning protocol for each group of athletes [10,14].

Moreover, substantial differences have been observed in the PAP interventions implemented in different studies. This great variability could explain the discrepancies observed in the degree of improvement attained by the athletes. In this respect, conflicting results have been found, including improvements in some studies and no effects or decreases in sports performance in other research [13]. Consequently, further research is required to determine which is the optimal PAP protocol for different groups of athletes and different sports disciplines because each type of exercise induces different effects, and the stimuli applied to young athletes differ from those of professional athletes [13,14].

As a result, it is necessary to clarify the effect of certain aspects that still remain unclear, such as the appropriate conditioning activity intensity, the number of sets to be performed, type of exercise used, and rest period between the conditioning stimulus and the activity [15,16]. The adequate dosage of these parameters has not been standardized in previous research [9]. In this regard, Seitz and Haff indicate that stronger athletes show greater PAP responses with shorter rest periods between the PAP stimulus and the activity, whereas weaker athletes would need a longer rest. They also stated that maximum loads induce better PAP responses in stronger athletes and submaximal loads in weaker athletes [13]. Weaker individuals obtain better PAP effects with multiple sets [13,17]. However, this could increase fatigue [10]. As for the type of exercise included in the conditioning activities, the squat (SQ) is commonly used to improve jumping ability since athletes have to apply strength in the vertical vector [13,17]. The SQ also has a greater range of motion than the hip thrust (HT), which can result in greater muscle tension [18]. However, in the case of running, the hip thrust (HT) exercise (which involves greater activation of the hip extensor muscles) has higher specificity and transferability to activities that require applying strength on a horizontal vector, such as sprinting [19,20,21]. In tennis, to our knowledge, only one recent study conducted by Terraza-Rebollo and Baiget has analyzed the effects of PAP [22]. This study verified that a PAP intervention did not affect serve velocity and accuracy in young competition tennis players. Likewise, no studies analyzing the influence of PAP on sprint performance in tennis players have been conducted. This ability, as previously mentioned, is a key performance factor in tennis. Additionally, since stronger athletes obtained greater PAP effects than weaker subjects in previous studies, it is necessary to find suitable protocols for the weaker athletes to attain acute performance improvements. Therefore, further research is warranted to verify the effects of PAP on key performance skills and abilities in tennis.

Moreover, it is essential to use quality assessment instruments to assess the improvements obtained in sprinting. For this purpose, the force–velocity (F–V) profile provides valuable information about the relationship between the force applied by one athlete and the speed at which his or her neuromuscular system generates it in ballistic or explosive movements (i.e., sprinting and running) performed with his or her lower limbs [23,24]. The F–V is calculated by a linear regression over a distance of 30 m [25], and it has proven to be reliable in youth athletes [26]. The profile is composed of different variables, some of them related to the sprint acceleration ability, such as maximum theoretical force (F_0_), maximum power (P_max_), and the maximal ratio of horizontal-to-resultant force (RF_peak_) [23,27].

In this context, the objective of the present study was to compare the acute effects of performing a full SQ and HT using two different loads (60% and 85% 1 RM) on sprinting ability in 19 junior male tennis players. The F–V was used to estimate the potential improvements obtained through the PAP. We hypothesized that both SQ and HT would effectively improve the sprint F–V profile and that more significant improvements would be obtained with loads of 60% 1 RM rather than 85% 1 RM.

## 2. Materials and Methods

### 2.1. Participants

Nineteen male tennis players of Benicarló Tennis Club (Castellón, Spain), with a minimum of three years of tennis training experience (4.47 ± 1.54) but without resistance training experience participated in the present study. Subjects´ characteristics are shown in Table 1. All study participants underwent an annual medical examination in the health services of the Valencian Tennis Federation, and none of them presented any injury or health condition that could prevent them from participating in this research. Study participants and their parents or guardians received detailed verbal and written information about the experimental protocol and the potential risks and benefits of participating in it. They were also allowed to withdraw from the study at any stage without penalty. All participants´ parents or guardians gave their written informed consent to be included in this research. The present study was conducted in accordance with the Declaration of Helsinki Ethical Principles. It was also approved by the Institutional Review Board of the Bio-ethics Committee at Prince Sultan University (Riyadh, Saudi Arabia) (ethical clearance number: PSU IRB-2021-02-0070).

### 2.2. Procedures

Before proceeding with data collection, anthropometric variables were recorded in the laboratory. Height and body mass were measured to the nearest 0.1 cm and 0.1 kg, respectively. Both height and body mass were measured with a digital measuring station—Seca 284, Hamburg (Germany). The experimental sessions were carried out always at the same time of the day (between 4.00 and 6.00 p.m.) to avoid the possible effect of circadian rhythm, and also because the participants´ training sessions were usually conducted at that time. The sessions were separated by a minimum of 72 h of rest time to avoid the impact of fatigue on speed test results. The tests were preceded by a 20 min warm-up consisting of seven minutes of jogging at a self-selected pace, eight minutes of dynamic stretching, and five minutes of progressive sprint bouts, with and without change of direction (60%, 70%, and 85% of perceived maximum). The participants performed maximal-effort 30 m linear sprints on a synthetic outdoor track. A smartphone application (My Sprint, Apple Inc., Cupertino, CA, USA) was used to record and analyze the trials’ split times (5, 10, 15, 20, 25, and 30 m). The recording was conducted with an iPhone 7 (iOS 10.0.2), mounted on a tripod, and located 10 m perpendicular to the sprint direction, just in front of the 15 m marker. The system is based on high-speed video analysis (240 frames per second), and it has proven to be valid and reliable to assess linear sprint performance in relation to two different reference systems: timing photocells and radar gun [25]. Subjects started from a crouching position (staggered-stance) with their right hand on the track. The beginning of the sprint was set when the right thumb of the athlete left the ground (this was detected by visual inspection with MySprint). Two independent observers were asked to select the first frame in which participants’ right thumb left the ground (i.e., the start of the sprint) and, subsequently, the frame in which the pelvis was aligned with each of the three different markers for each of the recorded sprints [23]. Split times, participants’ body mass, and height were used by the MySprint app to calculate F_0_, P_max_, and RF_peak_ following previously validated formulas [23,24,25].

### 2.3. Familiarization and Maximal Dynamic Strength Test

During the four weeks prior to the study commencement, 12 familiarization sessions were carried out to ensure the proper technique in the full SQ and HT exercises. These familiarization sessions consisted of four sets of seven, five, and three repetitions with loads of 60%, 70%, and 85% 1 RM, respectively, for the full SQ and HT exercises. The encoder Speed4Lifts (v2.0., Speed4Lifts, Madrid, Spain) was used to calculate the peak dynamic force, which uses the load–velocity relationship evaluation method, which involves measuring concentric velocity with two different weight loads, and then, through linear regression equations, it predicts the load (1 RM percentage) from velocity data [28,29]. All reported repetition velocities in this study correspond to the mean propulsive velocity (MPV) of the concentric phase [29]. The MPV was used in the present study for the following two reasons: (i) it has been proven to have a very high intra-and inter-participant reliability, similar to mean and peak velocity [30]; (ii) regarding the mean values of the propulsive phase (i.e., MPV), when assessing the velocity with which a load is lifted in a concentric action, it avoids underestimating individuals´ neuromuscular ability, especially when lifting light and medium loads [31].

### 2.4. Full Squat and Hip Thrust

The full SQ was performed starting from the upright position with the knees and hips fully extended. Each participant descended in a continuous movement until his upper thighs were below the horizontal plane and then immediately ascended back to the upright position. Participants were always required to execute the concentric phase of full SQ at maximal velocity. An SQ rack Fitness Line (Collado-Villalba, Spain) and a standard Olympic bar and weight plates (Eleiko, Halmstad, Sweden) were used for all sessions. To perform the HT, subjects were instructed to start by sitting on the ground with their legs flat on the floor, feet shoulder-width apart, and their upper back against a padded exercise bench. Using the same Olympic bar and disks utilized in the previous exercise, the bar was covered with a pad for comfort, and it was placed above the participants´ lower legs, slightly below their knees [32]. Once the subjects positioned the barbell above their pelvis, they assumed the starting position of the exercise by bringing their heels toward the bench and bending their knees. Then, subjects lifted their hips until their knee joints formed a 90° angle with their tibias.

### 2.5. Methodology

**Figure 1:**
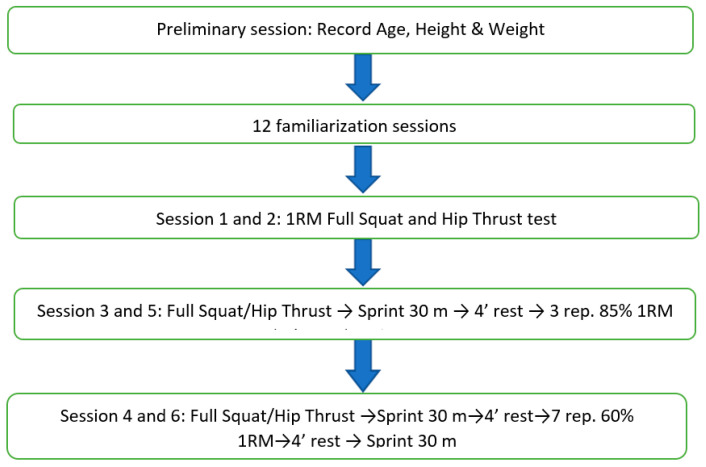
Description of the intervention process and the activities performed in each session.

The activities carried out during the intervention process are shown in Figure 1. In session 1, the 1 RM full SQ test was performed, and one week later, in session 2, the 1 RM HT test was conducted. A random selection of the participants was used. Thus, subjects were assigned to four groups, so that on each day, one group performed one type of exercise with a different load to avoid the learning effect, which could represent a threat to internal validity. All tests were performed at an outdoor facility maintained at standard environmental conditions. To simulate an “active” athletic setting, instead of seating during the rest period, the tennis players were instructed to perform an active recovery with short displacements in different directions at low intensity. Thirty seconds before testing, athletes were notified to be prepared. Session 3 began with the warm-up explained in Section 2.2. Subsequently, the 30 m sprint test was performed. Then, study participants rested for four minutes and performed three repetitions at 85% 1 RM of a full SQ, and after resting for four minutes, they performed the 30 m sprint test again. The session structure used in session 3 was applied in sessions 4, 5, and 6, but using a different conditioning stimulus. Thus, session 4 began 72 h later than session 3, and the activation protocol consisted of performing one set of seven repetitions of a full SQ at 60% of 1 RM. Session 5 started 96 h later, and the conditioning stimulus was one set of three repetitions of an HT at 85% of 1 RM. Finally, session 6 was performed 72 h later, and the activation protocol was one set of seven repetitions of an HT at 60% 1 RM. Moreover, the MySprint application was used to measure the F–V profile when the 30 m tests were performed.

### 2.6. Statistical Analysis

Data are presented using the format of the mean SD (standard deviation). The Shapiro–Wilk test was used to contrast the normality of the variables. To determine the consistency between the measurements made in the pre- and post-test, the interclass correlation coefficient (ICC) was calculated for all the assessed parameters. ICC values were interpreted as follows: ICC ≤ 0.49, poor; ≥ 0.50 ICC < 0.75, moderate; ≥0.75 ICC < 0.9, good; ICC ≥ 0.9, excellent (Koo and Li, 2016). To verify whether there were differences between groups in the baseline, a one-way ANOVA test was conducted. Furthermore, to assess the effects of PAP on the three different conditions (time: pre- vs. post-test; load: 85% vs. 60%; and exercise: HT vs. SQ), a factorial repeated measures ANOVA (2 × 2 × 2) was conducted. When statistically significant *p* values were found (interaction effects or significant main effects), a post hoc pairwise comparison was conducted with Sidak correction to identify those differences. The effect size was calculated using the partial eta squared (η^2^p). Values of η^2^p = 0.01, η^2^p = 0.06, and η^2^p = 0.14 were considered as small, medium, and large effect sizes, respectively [33]. The level of significance established was *p* = 0.05. The statistical analysis of the data was performed using the program IBM SPSS V.26^®^ computing (IBM Corp., Armonk, NY, USA).

## 3. Results

The results obtained by the subjects in the 1 RM tests are shown in Table 2, and the assessed F–V variables are presented in Table 3. The ICC values between test and retest were higher in all cases than 0.9, which reflects excellent reliability. In addition, the one-way ANOVA confirmed the absence of significant differences between the four different measurements conducted at the baseline. 

The 2 × 2 × 2 ANOVA confirmed the absence of interaction effects for all the assessed variables. However, a main effect of time was found for the following parameters: 5 m (F1–18 = 7.35; *p* = 0.014; 268 η^2^p = 0.290), 10 m (F1–18 = 8.62; *p* = 0.009; η^2^p = 0.324), P_max_ (F1–18 = 7.22; *p* = 0.015; η^2^p = 0.286), and RF_peak_ (F1–18 = 8.36; *p* = 0.010; η^2^p = 0.317). A main effect of exercise was also found for F_0_ (N) (F1–18 = 7.71; *p* = 0.035; η^2^p = 0.223). The results of the pairwise comparisons are shown below:

### 3.1. 5 m

Significant differences were observed after applying the PAP load of seven reps at 60% 1 RM in HP (*p* = 0.018; 95%CI = 0.010 to 0.090), and in SQ (*p* = 0.006; 95%CI = 0.015 to 0.78).

### 3.2. 10 m

A significant difference was found in the SQ (*p* = 0.003; 95%CI = 0.023 to 0.101) after applying the PAP load of seven reps at 60% 1 RM.

### 3.3. F_0_ (N)

A significant difference was observed between the HT and SQ in favor of the latter condition (*p* = 0.024; 95%CI = −0.415 to −0.033) after applying the PAP load of three repetitions at 85% 1 RM.

### 3.4. P_max_ (Wkg-1)

Significant differences in the HT (*p* = 0.013; 95%CI = −98.05 to −13.01) and in SQ (*p* = 0.037; 95%CI = −205.92 to −7.06) were found after applying the PAP load of seven reps at 60% 1 RM. 

### 3.5. RF_peak_

Significant differences were observed in HP (*p* = 0.016; 95%CI = −3.10 to −0.36), and in SQ (*p* = 0.007; 95%CI = −3.04 to −0.54) after applying the PAP load of seven reps at 60% 1 RM.

## 4. Discussion

The main finding of the present study was that the PAP protocol applied was effective in improving the 5 m time with 60% 1 RM using both an HT and SQ with an ES of 0.43 and 0.31, respectively. The PAP was also effective in improving 10 m times when the SQ was applied with a load of 60% 1 RM (ES = 0.34), confirming our hypothesis that moderate loads are sufficient to produce performance improvements in young athletes without previous strength training experience.

PAP has proven to be an effective warm-up to enhance maximal strength and speed of strength development [9,34]. In the present study, significant differences with a small effect size were found when the study participants performed the PAP with loads of 60% 1 RM using both the HT and SQ. These findings indicate that moderate loads may suffice in this population to enhance motor unit recruitment and synchronization, which are the main mechanisms associated with improved performance when applying PAP protocols [9]. The mentioned results were somewhat expected. Indeed, in previous studies, light stimuli applied to young athletes with little or no strength training experience significantly enhanced sprint performance by eliciting lower fatigue [13]. In this regard, a recent meta-analysis that included 32 primary studies showed greater effects (ES = 1.06) with moderate loads (60 to 84% 1 RM) than with high loads (ES = 0.31) (>85% 1 RM) [35] in 141 subjects aged 20 ± 5 years [20]. Likewise, it was observed that an HT is effective in 5 m, but its effect declines in 10 m. We consider that the HT is more effective in improving sprinting ability over shorter distances due to the nature of this exercise because, because unlike SQ, it mainly activates the hip extensor muscles [20]. Some authors also attributed the effectiveness of an HT in the first sprinting meters to the application of strength in the horizontal vector [20,21]. However, Fitzpatrick et al. state that this theory is flawed [36]. They argue that, according to the principle of dynamic correspondence, the forces applied by an athlete must be considered in relation to the coordinate system set by the athlete. While it is true that during accelerations, athletes apply force basically in the horizontal plane, that is because their bodies lean forward, meaning that the direction of the force applied both in accelerations and high-speed running is basically the same [36]. By contrast, SQ was effective both in 5 m, 10 m, and 30 m. In this case, the improvement could be attributed to the enhanced intramuscular coordination by increasing eccentric strength in extensor muscles, which results in a decreased ground contact time and consequently improves their stride frequency (i.e., V0) [19]. Based on the results, we interpret that SQ presents great levels of transferability to all sprinting phases.

The study results revealed the absence of significant differences in sprinting performance after applying the PAP using both the HT and SQ with a load of 85% 1 RM. One possible explanation is that the accumulated fatigue could have overridden the PAP effect [9]. It is plausible that applying heavy loads to subjects without previous strength training experience produces a degree of fatigue that prevents them from optimizing sprint performance. However, it must be taken into account that fatigue can result from using high-intensity conditioning stimuli but could also be due to factors such as excessive volume, short recovery, strenuous warm-up, or personal characteristics of each subject. In this regard, it should be noted that greater benefits have been observed after applying PAP protocols in trained subjects [13,35,37]. Another possible explanation of these results could be that the age of the subjects (15.61 ± 1.35) coincides with the mid-PHV (peak height velocity) [37]. At this stage, the natural growth of the adolescents can lead to poorer training results due to the temporary disruption in basic motor skills caused by the accelerated growth of long bones [38]. This stage is known as adolescent awkwardness [39] and impacts neuromuscular function and physical performance [38].

In the present study, the deep SQ was performed as it generates greater motor unit activation and synchronization [13]. In this regard, the study results also show that the subjects improved the F_0_ and P_max_ variables, which coincides with other studies where a deep SQ was also performed [14,17]. However, Seitz et.al. [13] obtained greater performance improvements using a half SQ (ES = 0.58) than full SQ (ES = 0.25). This discrepancy suggests that a deep SQ produces higher levels of acute fatigue [19] and therefore PAP become less effective. Even so, certain discrepancies have been found in this regard in the scientific literature since some authors did not observe significant differences in sprint performance after using either a half SQ or full SQ [20,21,22]. Therefore, it is not possible to determine the effect of PAP by performing a half SQ instead of full SQ. In this sense, it should be noted that the acute performance improvements that can be obtained depending on SQ depth vary according to the athlete’s level. Stronger subjects perform better with shallow SQs, whereas subjects with lower strength levels obtain greater improvements with deep SQs [3]. For this reason, the full SQ exercise was selected in the training protocol of the current research.

As for the F–V profile components, a significant increase in F_0_ after performing a SQ with a load of 85% was observed. Additionally, the P_max_ and RF_peak_ were also significantly increased after performing a SQ and HT with a load of 60% 1 RM. Improving the last two variables is crucial, since acceleration in short distances is a key performance factor in tennis. These results again suggest that both HT and SQ are effective in improving various F–V profile components, and the most appropriate intensity for both exercises is 60%, rather than 85%. Therefore, this reinforces the idea that in youth athletes without previous strength training experience, moderate loads should be used in the PAP protocols. 

Importantly, Seitz et al. [13] verified that resting time duration used after the conditioning activity should be set depending on the subject’s maximal strength. Thus, the PAP effect is greater in stronger subjects when short recovery times are used (i.e., 2–3 min), whereas longer rest intervals produce greater improvements in individuals with lower strength levels. This is because of the ratio of type II muscle fibers [40,41], which in turn is associated with a higher myosin light chain phosphorylation and represents the peripheral factor on which the PAP effects are based [42]. In our study, the resting time was four minutes. This selection is justified based on the results of previous studies [43,44,45]. However, it cannot be ruled out that a more extended recovery period could have produced lower fatigue to study participants, and consequently, they could have obtained better results. In this sense, Wilson et al. [35], after conducting a meta-analysis, verified that long rest periods (i.e., 7 to 10 m) could be more effective than short periods (3 to 7 min) (ES = 0.54 vs. 0.14).

Finally, only one set of HTs or SQs was undertaken in the conditioning protocol. However, some studies found greater effects of PAP when more than one set was performed (ES = 0.69 vs. ES = 0.24) [13,35,37], particularly in stronger athletes. Therefore, it is also possible that further PAP enhancements could have been attained if more than one set had been performed. However, it should also be considered that the study participants had no previous strength training experience. That means that performing more sets could have caused them great fatigue, as Wilson et al. state [35].

This study has some limitations that must be mentioned. The muscle activity was not measured. Thus, the results and conclusions are exclusively based on the PAP effects. Moreover, we must consider that, since the study participants knew the study’s objective, the occurrence of a placebo effect cannot be excluded. Future research should include control groups and verify the effect of performing full vs. half SQs and the results of using a different number of sets and different rest periods between the conditioning activity and the task.

## 5. Conclusions

The PAP protocols applied to junior tennis players without previous strength training experience effectively improved the F–V profile when the loading intensity used in the conditioning activity was 60% 1 RM and the exercises performed were either an HT or SQ. However, 85% 1 RM loads were not adequate to increase acute sprinting performance in this population group. In addition, HT presented a higher level of transferability in the first 5 m of sprinting, whereas SQ provided acute improvements in different sprinting phases. 

### Practical Applications

Both the HT and the SQ are used in sports training to improve sprinting speed or other sport skills and fitness components. The present study verified that both exercises could be useful in PAP protocols aiming to enhance sprinting ability. Practitioners and trainers can use them as a suitable PAP stimulus to induce acute effects on subsequent ballistic or explosive activities. However, the greatest effect occurs when moderate loads are applied in youth tennis players without previous strength training experience (60% 1 RM). Therefore, heavy loads may reduce their adaptative reserve prematurely and limit future performance improvements.

## Figures and Tables

**Table 1:** Participants descriptive information.

	Experimental Group (*n* = 19)
**Age (years)**	15.61 (1.35)
**Height (cm)**	173.89 (8.24)
**Weight (kg)**	68.31 (13.34)
**Tennis training experience (years)**	4.47 (1.54)

Note: Data is presented as mean (standard deviation).

**Table 2:** Results obtained by the study participants in the 1 RM tests.

Exercise	1 RM Test Result (kg)
Hip thrust	53.11 (15.71)
Squat	66.89 (17.16)

**Table 3:** Results obtained by the study participants in the force–velocity variables assessed before (pre-) and after (post-) applying the post-activation potentiation.

Moment	Results Obtained before Applying the Post-Activation Potentiation					
	5 m	10 m	30 m	F_0_ (N)	P_max_ (N)	RF_peak_
Pre-85%1 RM-HIP THRUST	1.62 (0.11)	2.4 3(0.15)	5.45 (0.39)	429.90 (128.82)	791.20 (274.11)	44.50 (4.13)
Pre-PAP-60%1 RM-HIP THRUST	1.60 (0.12)	2.41 (0.14)	5.40 (0.37)	441.90 (114.83)	818.20 (245.02)	45.23 (3.90)
Pre-PAP-85%1 RM-SQUAT	1.55 (0.12)	2.33 (0.16)	5.23 (0.41)	467.66 (120.11)	895.69 (268.72)	47.16 (4.33)
Pre-PAP-60%1 RM-SQUAT	1.60 (0.17)	2.42 (0.20)	5.42 (0.44)	430.47 (132.40)	797.86 (288.61	45.14 (5.65)
	Results obtained after applying the post-activation potentiation					
	5 m	10 m	30 m	F _0_(N)	P_max_ (N)	RF_peak_
Post-PAP-85%1 RM-HIP THRUST	1.59 (0.11)	2.40 (0.16)	5.42 (0.39)	448.90 (128.36)	824.79 (276.91)	45.44 (4.08)
Post-PAP-60%1 RM-HIP THRUST	1.55 (0.11) *	2.36 (0.17)	5.38 (0.45)	475.95 (124.44)	873.73 (285.38) *	46.97 (4.28) *
Post-PAP-85%1 RM-SQUAT	1.55 (0.12)	2.32 (0.15)	5.26 (0.39)	479.18 (127.05) *	904.35 (274.73)	47.53 (4.38)
Post-PAP-60%1 RM-SQUAT	1.55 (0.15) *	2.35 (0.20) *	5.32 (0.45) *	473.17 (147.74)	885.14 (329.37) *	46.94 (5.37) *

5 m: 5 m split sprint time; 10 m: 10 m split sprint time; 30 m: 30 m split sprint time; F_0_(N): maximal theoretical velocity; P_max_ (N): maximal power; RF_Peak_ (%): maximal ratio of horizontal-to-resultant force; PAP: post-activation potentiation; 1 RM: 1 maximum repetition; *: significant effect found (*p* < 0.05).

## Data Availability

Not applicable.
